# Light Fractionation Significantly Increases the Efficacy of Photodynamic Therapy Using BF-200 ALA in Normal Mouse Skin

**DOI:** 10.1371/journal.pone.0148850

**Published:** 2016-02-12

**Authors:** Henriëtte S. de Bruijn, Sander Brooks, Angélique van der Ploeg-van den Heuvel, Timo L. M. ten Hagen, Ellen R. M. de Haas, Dominic J. Robinson

**Affiliations:** 1 Center for Optical Diagnostics and Therapy, Department of Otolaryngology and Head & Neck Surgery, Erasmus MC, Rotterdam, The Netherlands; 2 Department of Dermatology, Erasmus MC, Rotterdam, The Netherlands; 3 Department of Surgical Oncology, Erasmus MC, Rotterdam, The Netherlands; University of Aveiro, PORTUGAL

## Abstract

**Background:**

Light fractionation significantly increases the efficacy of 5-aminolevulinic acid (ALA) based photodynamic therapy (PDT) using the nano-emulsion based gel formulation BF-200. PDT using BF-200 ALA has recently been clinically approved and is under investigation in several phase III trials for the treatment of actinic keratosis. This study is the first to compare BF-200 ALA with ALA in preclinical models.

**Results:**

In hairless mouse skin there is no difference in the temporal and spatial distribution of protoporphyrin IX determined by superficial imaging and fluorescence microscopy in frozen sections. In the skin-fold chamber model, BF-200 ALA leads to more PpIX fluorescence at depth in the skin compared to ALA suggesting an enhanced penetration of BF-200 ALA. Light fractionated PDT after BF-200 ALA application results in significantly more visual skin damage following PDT compared to a single illumination. Both ALA formulations show the same visual skin damage, rate of photobleaching and change in vascular volume immediately after PDT. Fluorescence immunohistochemical imaging shows loss of VE-cadherin in the vasculature at day 1 post PDT which is greater after BF-200 ALA compared to ALA and more profound after light fractionation compared to a single illumination.

**Discussion:**

The present study illustrates the clinical potential of light fractionated PDT using BF-200 ALA for enhancing PDT efficacy in (pre-) malignant skin conditions such as basal cell carcinoma and vulval intraepithelial neoplasia and its application in other lesion such as cervical intraepithelial neoplasia and oral squamous cell carcinoma where current approaches have limited efficacy.

## Introduction

Photodynamic therapy (PDT) using topically applied 5-aminolevulinic acid (ALA) or other protoporphyrin IX (PpIX) precursors is used as a treatment modality for various (pre-) malignant skin lesions [1–2]. It is a widely approved therapy for actinic keratosis, squamous cell carcinoma in situ, superficial and certain thin basal cell carcinomas [2–3]. It is under investigation for the treatment of premalignant oral lesions [4–5] and several gynaecological pre-malignancies [6–8].

Although ALA-PDT is effective, it is predominantly applied for thin lesions and long-term responses can be improved. Different strategies have been investigated to improve the results such as ALA penetration enhancers, the use of ALA ester derivatives, certain physical methods such as tape stripping and iontophoresis and the development of carriers such as liposomes or nanoparticles [9–11]. A alternative method to improve the efficacy is to modulate the haem synthesis pathway so that higher concentrations of PpIX are accumulated. This can be achieved by use of iron chelation or vitamin D administration [12–13].

An approach we have been investigating is the used of light fractionation with a single long dark interval. We have shown that light fractionation significantly increases the effectiveness of ALA-PDT both in preclinical and clinical studies [14–19]. The complete response rate of superficial basal cell carcinomas 5 years after treatment with light fractionated ALA-PDT is 88% compared to only 75% after exposure to a single fraction [19]. In several studies we have optimized the parameters for light fractionation and found that a 2 hour dark interval between two light fractions of which the first fraction is relatively small compared to the second fraction was the most optimal treatment scheme [20–21]. It is important to note that for the two most important ALA-esters; methyl aminolevulinate (MAL) and hexaminolevulinate light fractionation did not result in a significant increase in efficacy that is observed using ALA [22–23].

The mechanism responsible for the increased effectiveness of light fractionated ALA-PDT is not simply the result of the utilization of re-accumulation of PpIX during the dark interval between the two light fractions [21]. Furthermore, the acute immune response only plays a bystander role [24]. Our current hypothesis is that cells become sublethally damaged during the first fraction which renders them more vulnerable to a second light fraction 2 hours later [25]. The unexpected difference in response to light fractionation between ALA and MAL was used to further explore the mechanism. An important difference between the use of ALA and MAL is the spatial distribution of PpIX in mouse skin. Moan *et al*. showed that ALA penetrates more easily into the circulation whereas MAL remains at the site of application [26]. We have previously shown that more endothelial cells accumulate a higher concentration of PpIX after ALA than after MAL application and ALA-PDT results in more vascular damage than MAL-PDT [27]. Based on these results it is clear that the spatial distribution and in particular endothelial accumulation of PpIX is an important factor for the increased response to light fractionation.

BF-200 ALA, a recently approved nanoemulsion-based gel formulation containing 7.8% ALA (10% ALA hydrochloride), is under investigation in several phase III trials for the treatment of actinic keratosis (AK) [28–29]. In phase III trials BF-200 ALA shows slightly lower recurrence rates for AK compared to MAL-PDT with 6–12 months follow-up [28]. In a preclinical study Maisch et al. showed deeper penetration of BF-200 ALA compared to MAL in ex-vivo pig skin [30]. These results encouraged us to investigate light fractionation with this new formulation of ALA. An optimized light fractionation scheme using BF-200 ALA could potentially lead to enhanced clinical responses in thicker lesions in the skin and in other more difficult to treat lesions in the oral cavity, and in the gynaecological tract.

The current study is the first to compare BF-200 ALA with ALA in preclinical models. We investigated the fluorescence kinetics of BF-200 ALA in normal hairless mice and compared that with ALA and MAL. We also investigated the fluorescence distribution both in-vivo and ex-vivo in pig and mouse skin. We investigated the PDT induced damage after BF-200 ALA and ALA using light fractionation and compared the visual skin damage and the vascular responses of mouse skin. If light fractionation can be shown to enhance the efficacy of PDT using BF-200 ALA there is the potential to adopt this approach in the clinic.

## Materials and Methods

### Experimental design

The animal ethics committee of the Erasmus MC approved the experimental protocols of the study. We investigated the pharmacokinetics of PpIX distribution using (multispectral-) fluorescence imaging and assessed PDT efficacy after the administration of different porphyrin precursors in a number of cutaneous murine and porcine models. An overview of these protocols and the number of animals in individual groups is shown in Table 1.

**Table 1:** Overview of numbers of mice (m) and pigs (p) used in-vivo or ex-vivo experiments.

Group	Species	In-vivo or ex-vivo	PpIX precursor				Fluorescence imaging		PDT/nm	PDT induced damage
			ALA	BF	MAL	Con	Location	Time/h		
1	m	In	6	6	6	6	Superficial	0–24	-	-
2	m	In	6	6	6	6	Superficial, Sections	4	-	-
3	m	Ex	6	6	6	6	Superficial, Sections	4	-	-
4	p	Ex	6	6	6	6	Superficial, Sections	4	-	-
5	m	In	6+6	6+6	-	-	Superficial, Bleaching	4+4&6	532	Visual
6	m	In	4+4	4+4	-	-	Superficial, Bleaching	4+4&6	532	Sections
7	m	In	5+5	5+5	-	-	Intra-vital	4+4&6	630	Intra-vital

For each experimental group the table shows the species, whether it was an in-vivo or ex-vivo experiment, the PpIX precursor that was applied (ALA, BF-200 ALA (BF), MAL or vehicle (Con)), at what time point (multispectral-) fluorescence imaging was performed from the surface, in sections or using intravital microscopy in the skin-fold window and, in case of PDT, the used illumination wavelength and the method of determining PDT induced damage (monitored for visual skin damage or vascular responses determined intra-vitally or in sections).

### Animal models

Four different animal models were used: the normal skin of female outbred hairless mice (SKH1-hr, Charles River, Someren, The Netherlands) aged between 8 and 10 weeks, the mouse skin-fold window chamber, ex-vivo porcine skin and ex-vivo murine skin [31–32]. The mouse skin-fold chamber was prepared on the back of SKH1-hr mice using a procedure adapted from a previous study [31]. Mice received analgesia 1 hour (1 mg/kg rimadyl cattle s.c; Pfizer, Capelle a/d IJssel, NL) and anaesthesia 20 minutes (75 mg/kg ketamine i.p.; Alfasan, Woerden, NL and 1 mg/kg medetomidine i.p.; Eurovet, Bladel, NL) before the skin-fold chamber operation procedure. The dorsal skin was folded and fixed between two frames after removal of one side of the skin in 1 cm diameter up to the fascia of the opposed skin. Glass spacers (thick cover glasses of 9 mm in diameter) were placed on the epidermal side of the skin and two 12 mm circular microscopic cover glasses were used to close the frames on both sides. Mice were housed individually in climate controlled cabinets with an ambient temperature of 30°C and a humidity of 70%. Experiments started 1 day after the preparation of the chambers. From two weeks prior to the experiments, all mice were fed a chlorophyll free diet (catalogue number 4208.00, Hope Farms b.v., Woerden, NL) to minimize the contribution of pheophorbides to the autofluorescence emission spectrum.

Ex-vivo porcine and murine skin was prepared as described previously [32]. Ex-vivo pig skin samples were collected from surplus pigs used in unrelated anatomical studies in the Erasmus MC. Ex-vivo mouse skin was harvested from control animals treated with carboxymethyl cellulose (group 2, Table 1). Areas of skin were removed immediately after sacrificing the pig or just before sacrificing the mouse and put on ice. Subcutaneous fat was removed and the skin was cut in ~1x1 cm pieces and placed in a Petri dish, dermis down, containing solid 5% agar medium (Sigma-Aldrich Chemie BV, Zwijndrecht, NL) with 145 mM NaCl (Calbiochem, Darmstadt, DE), 5 mM KCl, 10 mM HEPES, 10 mM glucose, 1 mM MgSO_4_ (Sigma-Aldrich) in distilled water.

### Porphyrin precursors

BF-200 ALA (marketed as Ameluz) a 10% aminolevulinic acid HCl nano-formulation, (Biofrontera AG, Leverkusen, Germany) and MAL (Metvix, 160 mg/g, Galderma, Freiburg, Germany) were supplied by the manufacturer ready-for-use. ALA was used in two formulations. To compare the pharmacokinetics of PpIX distribution after ALA and BF-200 ALA application (groups 1–4, Table 1) 10% ALA (Fagron, Capelle aan de IJssel, NL) was dissolved in 3% carboxymethyl cellulose (Erasmus MC Pharmacy, Rotterdam, The Netherlands) in water. NaOH was added to obtain pH4. For the PDT induced damage studies (group 5–7, Table 1) a clinical formulation of 20% ALA (de Magistrale Bereider, Oud-Beijerland, NL) was used [33].

### Superficial fluorescence kinetics in mouse skin

Superficial PpIX fluorescence images were recorded at 0, 2, 4, 6, 8, 10, 12 and 24 hours after precursor application (group 1, Table 1). The 2 hour measurement was performed by temporarily removing the occlusive dressing from the site of administration. Superficial fluorescence imaging was performed used a set-up adapted from that described previously using 532 nm excitation light and 625±20nm detection [23].

### PpIX fluorescence distribution and co-localisation with endothelial cells

The microscopic distribution of PpIX within the skin was investigated in skin samples harvested at 4 hours (group 2, Table 1), in ex-vivo skin samples applied for 4 hours (group 3 and 4) and intra-vitally in the skin-fold chamber model (group 7). Skin samples were snap frozen and stored at -80°C. Cross-sections of 50 μm were cut and mounted on glass slides (StarFrost, Waldemar Knittel Glasbearbeitungs, DE) and were stained for endothelial cells using anti CD31 alexaFluor488 on the same day to visualize PpIX and CD31 fluorescence as described previously [27]. The PpIX distribution at depth in the dermis was investigated using intra-vital confocal microscopy. The precursor was topically applied to the epidermal side of the skin-fold for 4 hours and intra-vital confocal images of the subcutaneous musculature and lower dermis were recorded under 2–3% Isoflurane in oxygen anaesthesia.

### PDT and visual skin damage

PDT efficacy was determined in SKH1-hr mice by visually scoring the response of skin to PDT (group 5, Table 1). PDT and fluorescence imaging during the illumination was performed using the superficial fluorescence pharmacokinetics measurement set-up. The total light dose and irradiance (100 J/cm^2^ at 50 mw/cm^2^) with 514 nm used in previous studies were translated to 532 nm based on the in-vivo absorption spectrum of PpIX in mouse skin and the photon energy [27]. This resulted in a light dose of 178 J/cm^2^ at a irradiance of 89 mW/cm^2^. PDT was performed in a single illumination delivered at 4 hrs or according to a light fractionation scheme where the illumination is performed 4 and 6 hours after the administration of porphyrin precursor. In the light fractionated scheme the light dose of the first fraction was based on the delivered PDT-dose i.e. the light dose that led to 48% PpIX photobleaching, which is considered to be the optimal light dose for the first fraction [20]. The PDT induced damage was scored under 2–3% Isoflurane in oxygen anaesthesia for up to 14 days after the illumination; daily for the first 7 days and 3 times in the last week. The damage was photographed and scored visually by HdeB, blinded from the treatment schemes, according to a zero to 5-point scale as described previously [34].

### PDT and vascular response

The vascular response to PDT was investigated intra-vitally using the skin-fold chamber model and ex-vivo in skin samples harvested 24 hours after PDT.

#### Ex-vivo vascular response

The PDT induced vascular damage was histologically investigated in SKH1-hr mice by collecting the skin in the illuminated area and of the contralateral side at day 1 after PDT (group 6, Table 1). The PDT illumination was performed as described above for the visual skin damage experiment. Skin samples were harvested 24 hours after PDT under 2–3% Isoflurane in oxygen anaesthesia before mice were sacrificed. Skin samples were snap frozen and stored at -80°C. For the ex-vivo mouse skin experiments, extra pieces of skin was harvested from the control mice and immediately used for that experiment (group 3, Table 1). Frozen skin was imbedded in Tissue Tek O.C.T.^™^ Compound (Sakura Holland B.V.) and 5 and 50 μm cross-sections were cut with a cryostat, mounted on glass slides (StarFrost, Waldemar Knittel Glasbearbeitungs) and air-dried. The 50 μm sections were used for fluorescence labelled IHC staining of CD31 and CD144 on the same day and the staining procedure was performed as described previously [27].

#### Intra-vital microscopy vascular response measurements

In a last group of SKH1-hr mice the vascular response to ALA-PDT and BF-200 PDT was investigated at depth in skin using the chamber model and intra-vital confocal microscopy (group 7, Table 1). Confocal images of the subcutaneous musculature and lower dermis were recorded before and after illumination using a Zeiss Laser Scanner Microscope 510 now equipped with a 10x Plan-Neofluar objective, heated stage and a gas anaesthesia supply unit. All measurements and PDT illumination was performed under 2–3% Isoflurane in oxygen anaesthesia.

PDT was performed using a 630 nm laser (Visuals 630, Carl Zeiss B.V. Sliedrecht, NL) and a microlens (Medlight SA, Ecublens, Switzerland). The total light dose and irradiance (100 J/cm^2^ at 50 mw/cm^2^) used in previous experiments with 514 nm were translated to 630 nm based on the in-vivo absorption spectrum of PpIX in mouse skin and the photon energy as described above. This resulted in a light dose of 130.6 Jcm^-2^ at 65.3 mWcm^-2^ either delivered in a single illumination or according to a light fractionation scheme. In this skin-fold window experiment photobleaching during PDT could not be monitored and the illumination of the first light fraction was standardized to be 6.53 Jcm^-2^, which is consistent with 5 Jcm^-2^ at 514 nm. The transmission images were also used to determine the vascular area pre and post PDT. The area of a vessel was measured by drawing regions of interest in the LSM aim software. While the animal is repositioned between measurements care was taken to measure the same length of vessel for each series of images pre and post PDT.

### Statistics

Results are shown as mean ± standard deviation unless mentioned otherwise. For data with a standard deviation the weighted mean and standard deviation was calculated for the group. The significance of differences in PpIX fluorescence intensity (determined at the surface) and distribution (co-localisation of PpIX with endothelial cells or intensity in depth in the skin-fold chamber) and PDT induced damage (using visual skin damage scores, rate of photobleaching or changes in vascular lumen) was determined using Student’s t test, ANOVA or SNK test and considered significant when *p*< 0.05.

## Results

### Comparing ALA and BF-200 ALA induced PpIX fluorescence kinetics and distribution

#### Surface fluorescence pharmacokinetic measurements in mouse skin

**Fig 1:**
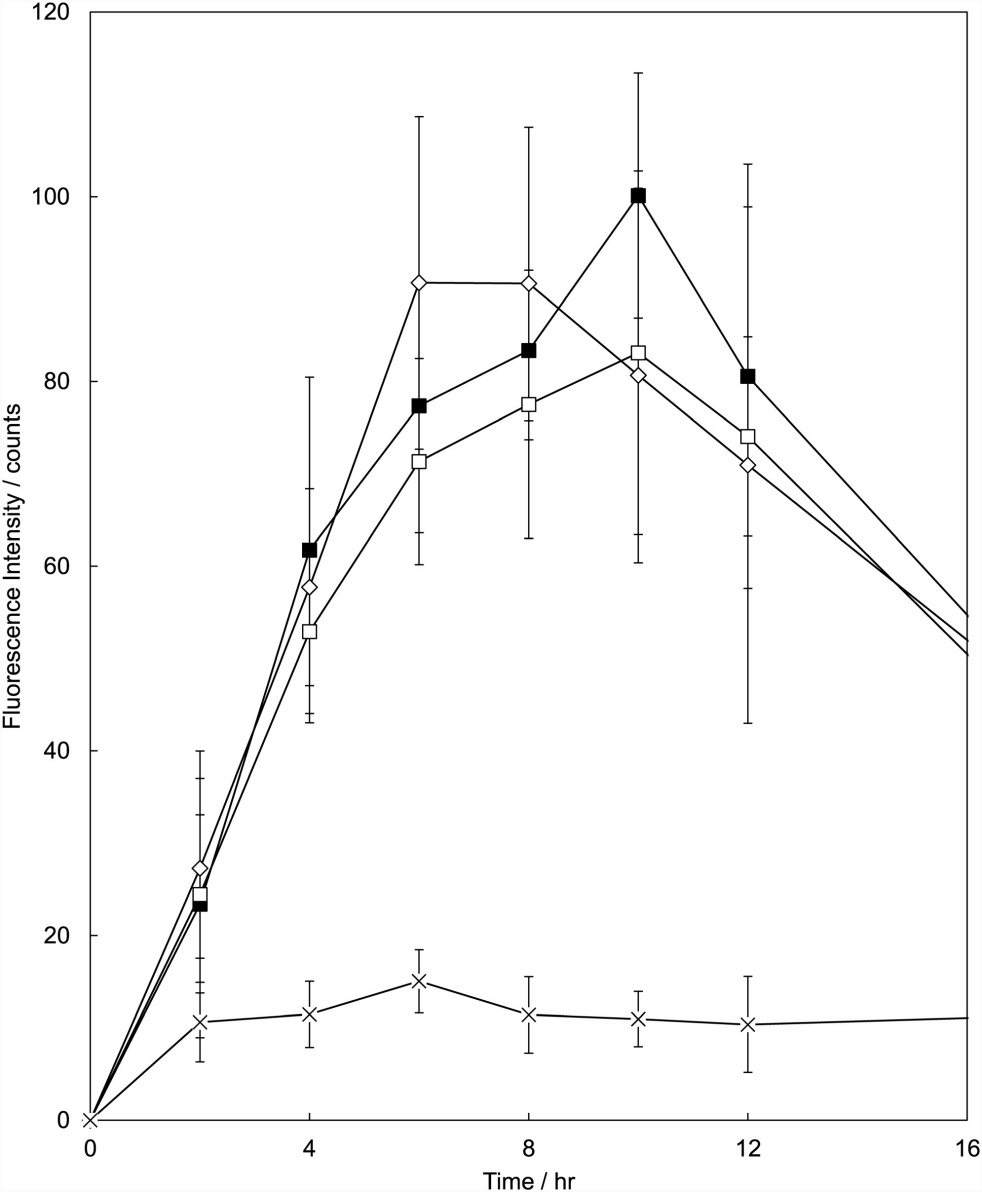
PpIX fluorescence kinetics of hairless mouse skin determined from superficial fluorescence imaging. The PpIX precursors were topically applied for 4 hours (ALA (■), MAL (◊), BF-200 ALA (□) and vehicle (x)). Values were corrected for dark current and individual autofluorescence. There is no statistically significant difference in area under the curve between the three precursors. Mean ± SD.

The PpIX fluorescence increase was similar up to 4 hours after topical application of ALA, MAL and BF-200 ALA (Fig 1). Between 4 and 12 hours the curves show slight differences. MAL peaks between 6 and 8 hours whereas ALA and BF-200 ALA show an increase in fluorescence up to 10 hours. At 24 hours the fluorescence intensity decreased to background levels again. The area under the curve calculated between 0 and 12 hours or between 0 and 24 hours was not significantly different between the 3 precursors (ANOVA, p = 0.568 and 0.738 respectively).

#### PpIX fluorescence distribution and co-localisation with endothelial cells

**Fig 2:**
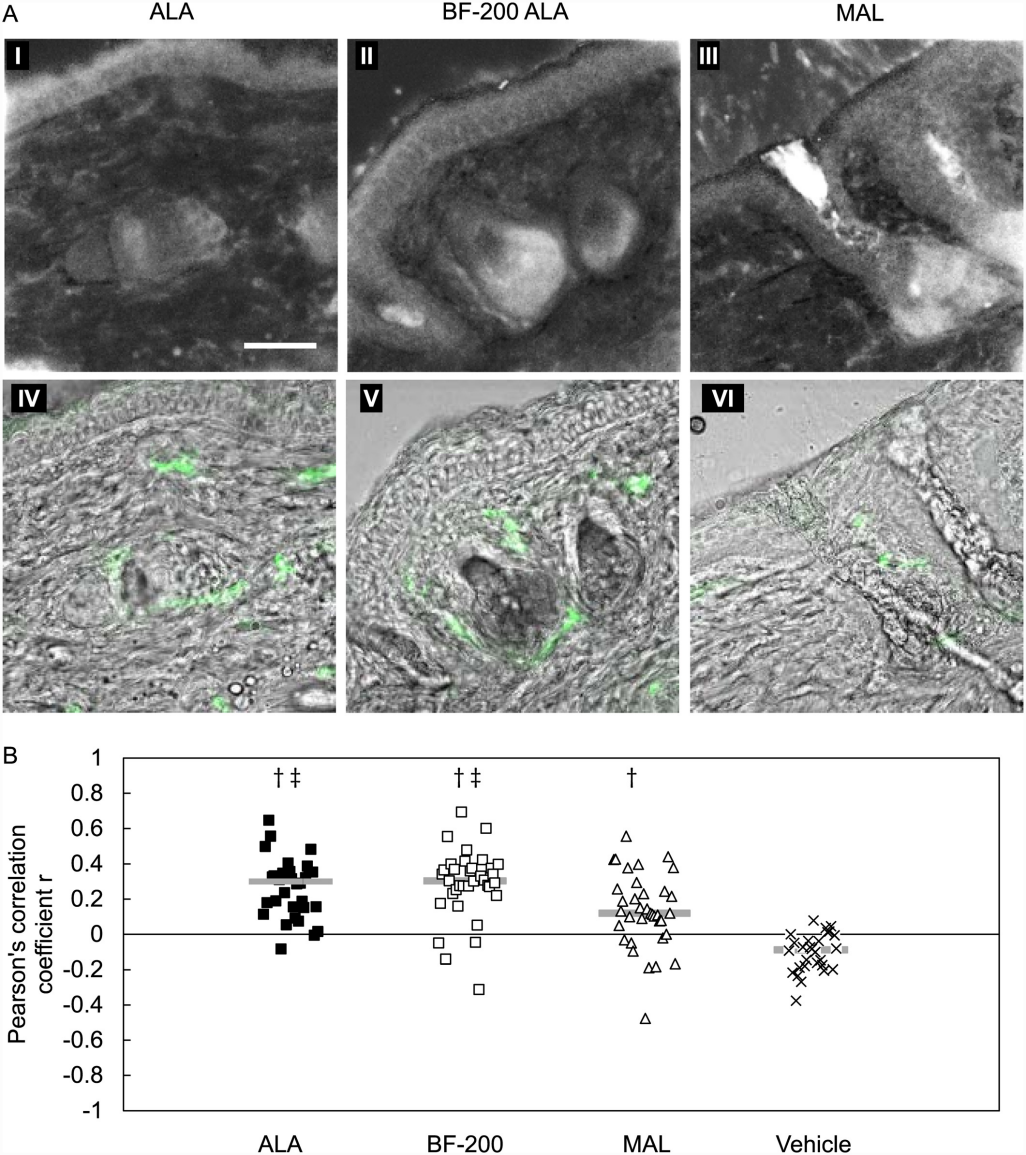
Hairless mouse skin sections with PpIX fluorescence stained for endothelial cells (CD31). A: PpIX fluorescence images (I-III) and the corresponding transmission-CD31 overlay images (IV-VI) of hairless mouse skin sections 4 hours after topical ALA, BF-200 ALA and MAL application. Bar = 50um. B: Pearson’s correlation coefficient r for PpIX and CD31 fluorescence determined in hairless mouse skin sections collected and stained 4 hours after topical ALA, BF-200 ALA (BF-200), MAL and vehicle application. Grey bar represents the median of the group (n = 28–36) where -1 represent complete exclusion and +1 complete co-localization. † statistically significant different from vehicle with p<0.01 (SNK-test), ‡ statistically significant different from MAL with p<0.01 (SNK-test).

Representative examples of confocal PpIX fluorescence images obtained are displayed in the left column of Fig 2A and the corresponding CD31 fluorescence and transmission overlay image is depicted in the right column. For all precursors high fluorescence intensities were observed in the epidermis, hair follicles and sebaceous glands. Also in the dermis fluorescent structures/cells was observed. Accumulation of PpIX in endothelial cells was determined by investigating the co-localization of the endothelial CD31 marker with PpIX fluorescence (Fig 2B). The mean Pearson’s correlation coefficient *r* after ALA application was not significantly different from BF-200 ALA. The correlation after MAL and vehicle showed both statistical significant differences with ALA and BF-200 and with each other (SNK test, p<0,01).

**Fig 3:**
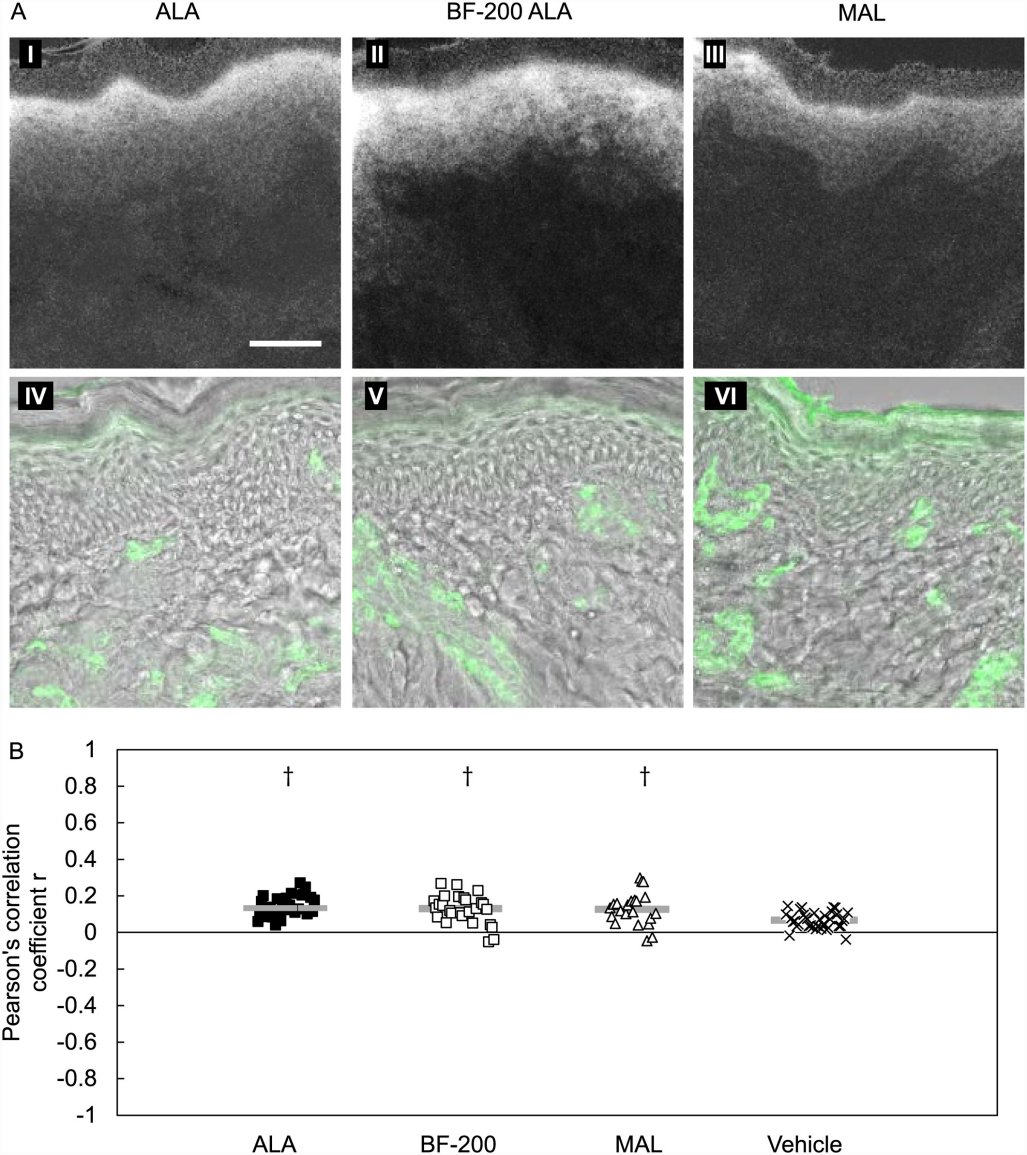
Ex-vivo pig skin sections with PpIX fluorescence stained for endothelial cells (CD31). A: PpIX fluorescence images (I-III) and the corresponding transmission-CD31 overlay images (IV-VI) of ex-vivo pig skin 4 hours after topical ALA, BF-200 ALA and MAL application. Bar = 50um. B: Pearson’s correlation coefficient for PpIX and anti-CD31 fluorescence determined in ex-vivo pig skin sections 4 hours after topical ALA, BF-200 ALA (BF-200), MAL and vehicle application. A value of -1 represent complete exclusion and +1 represents complete co-localization. † statistically significant different from vehicle with p<0.01 (SNK test).

The fluorescence distribution was also investigated in ex-vivo mouse and pig skin. Surface fluorescence measurements after 4 hours of topical application on the ex-vivo mouse skin showed almost no PpIX accumulation for any of the precursors investigated. The fluorescence distribution in these ex-vivo mouse skin samples was not investigated further due to the lack of fluorescence at the surface. On the contrary, in ex-vivo pig skin, 4 hours of topical precursor application resulted in detectable PpIX surface fluorescence intensities. An example of PpIX fluorescence images and the corresponding CD31 fluorescence images of ex-vivo pig skin samples is shown in Fig 3A. The epidermis showed high fluorescence intensities after all PpIX precursors investigated. After MAL application we observed a stronger demarcation from epidermis to dermis compared with ALA and BF-200 ALA. In all groups we found fluorescence in the dermis that showed some correlation with the CD31-stained endothelial cells. As in mouse skin, the mean Pearson’s correlation coefficient *r* after ALA application was not significantly different from BF-200 ALA (Fig 3B). In pig skin we were unable to show a difference between MAL and the two ALA formulations.

#### Intra-vital PpIX fluorescence distribution low in dermis

**Fig 4:**
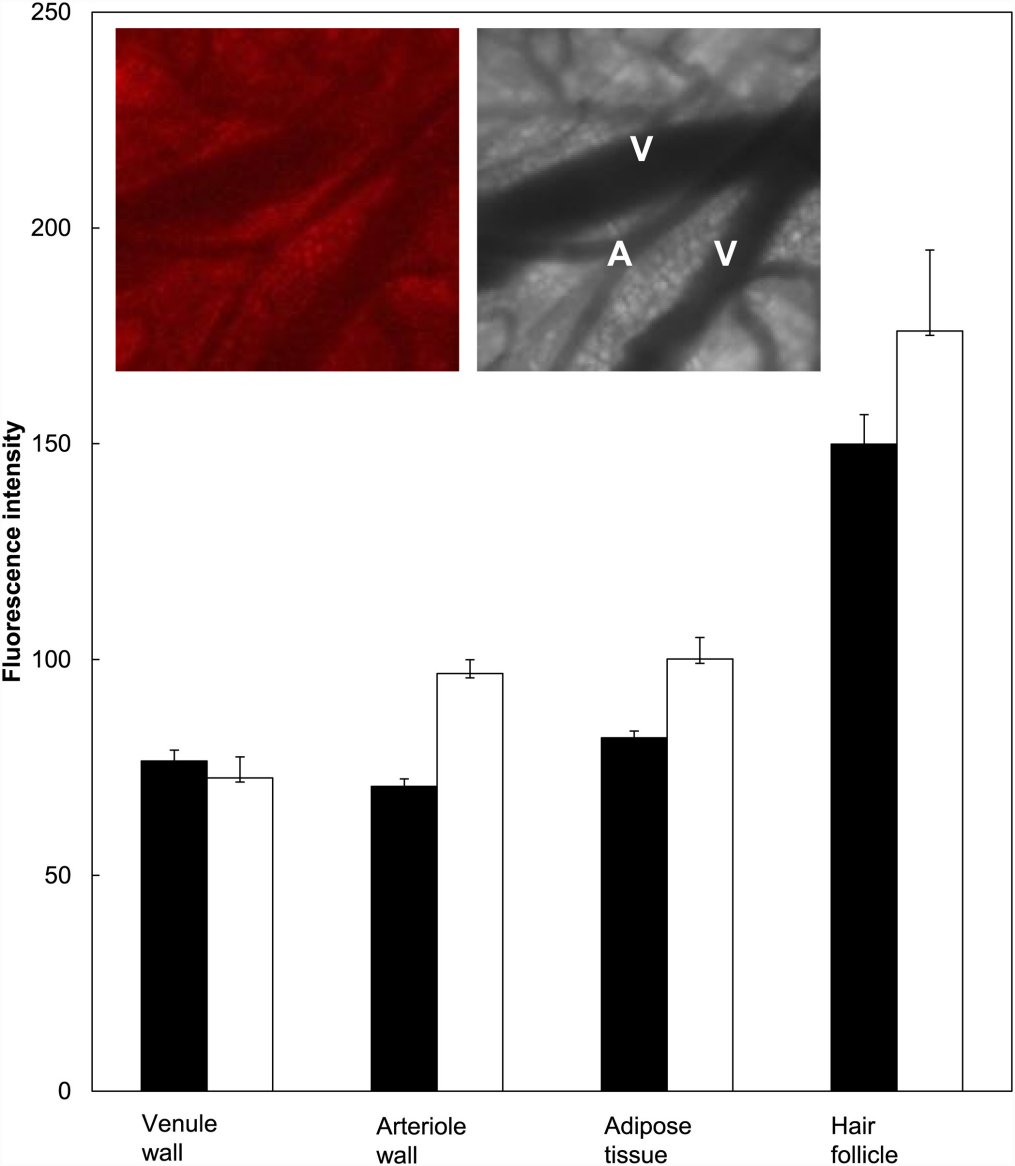
PpIX fluorescence determined using the intra-vitally skin-fold chamber model. Fluorescence intensity in different locations low in the dermis determined 4 hours after ALA (black) or BF-200 ALA (white) application using intra-vital confocal microscopy and the skin-fold chamber model. Insert shows an example of intra-vital confocal fluorescence microscopy and transmission images (V = venule, A = arteriole).

Intra-vital confocal microscopy of skin-fold chambers applied with ALA or BF-200 ALA showed high fluorescence intensities low in the dermis just above the subcutaneous musculature. An example of collected fluorescence and corresponding transmission images is shown in the insert of Fig 4. BF-200 ALA showed significantly more PpIX fluorescence than ALA in the arteriole wall, adipose tissue and/or sub cutaneous musculature and in hair follicles (weighted mean and SD, t-test, n = 10 animals, p<0.05).

### Comparing PDT induced damage after ALA or BF-200 ALA application

#### Visual skin damage

**Fig 5:**
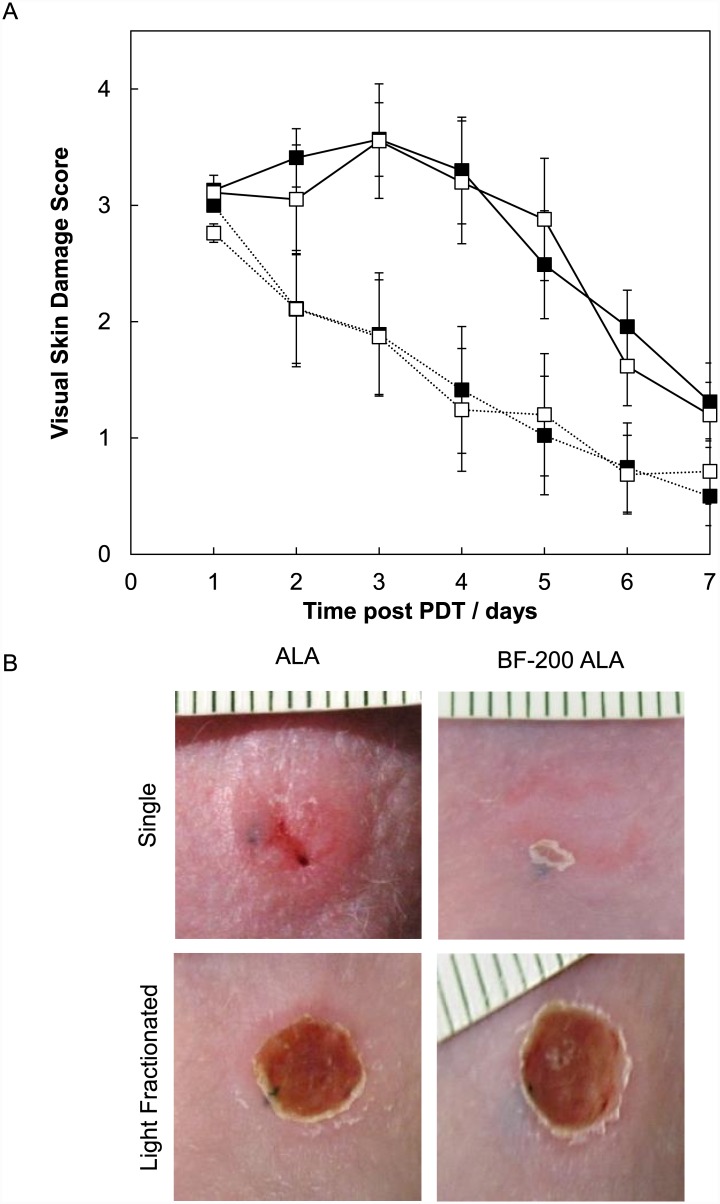
PDT induced visual skin damage in hairless mouse skin. A: The mean visual skin damage over time after single (dotted line) or light-fractionated PDT (solid line) using ALA (■) or BF-200 ALA (□). Mean ± SEM. B: Pictures of visual skin damage scores taken at day 4 after single or light-fractionated PDT using ALA or BF-200 ALA.

The mean visual skin damage scores after ALA-PDT were not different from those observed after BF-200 ALA-PDT, independent of the illumination scheme used (Fig 5A). Light fractionated illumination resulted in an increased visual skin damage score compared to a single illumination after both ALA and BF-200 ALA (t-test, p = 0.012 and 0.083 respectively). For BF-200 ALA-PDT this was not statistically significant due to one animal that showed unexpected high damage in the single illumination group. In general, skin treated with light fractionation showed a larger area with crust formation for a prolonged period after PDT whereas a single illumination in some cases did not lead to crust formation at all. Fig 5B shows a set of pictures of the visual skin damage at day 4 after single or light-fractionated PDT using ALA or BF-200 ALA.

**Fig 6:**
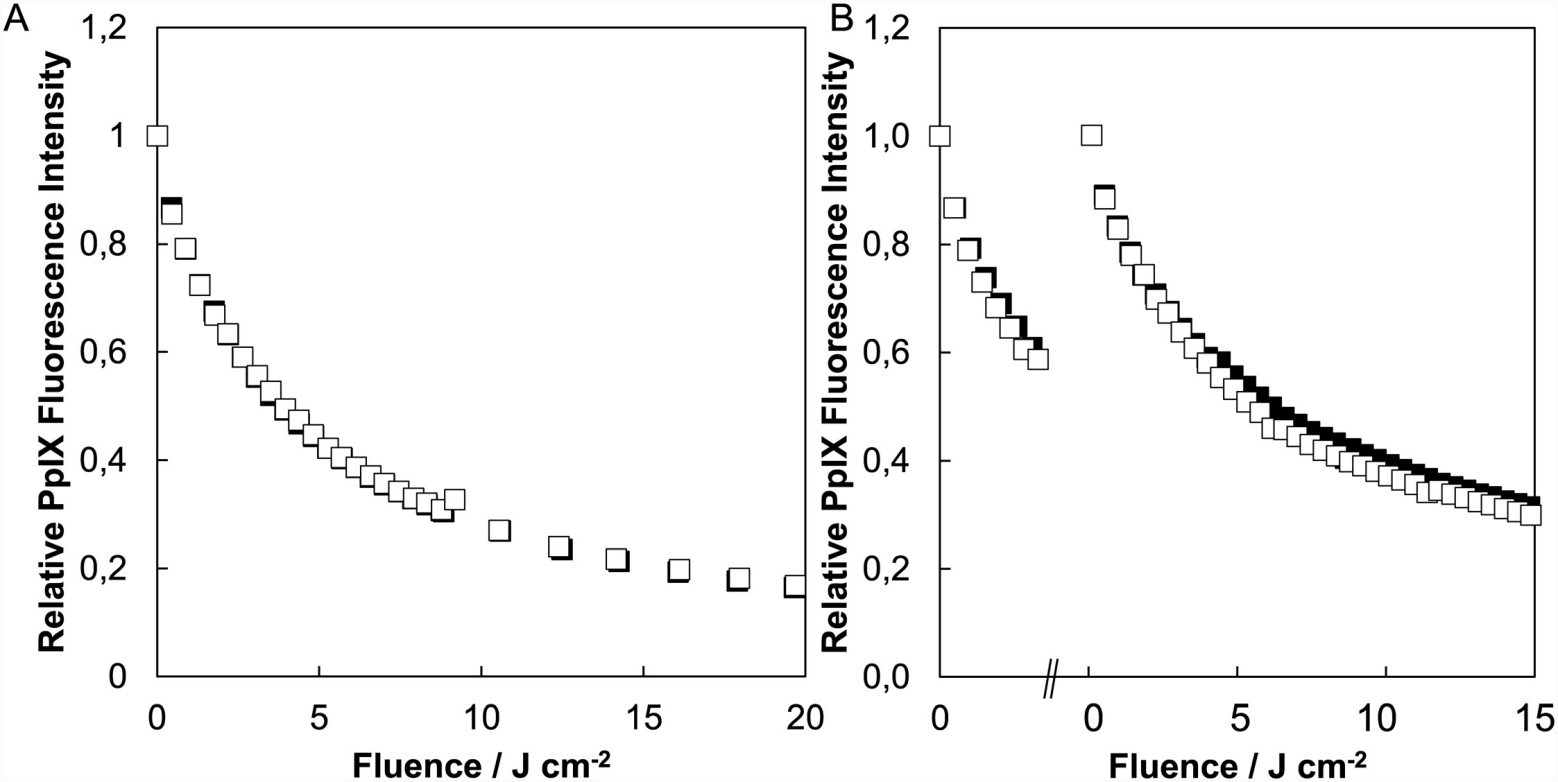
PpIX photobleaching during PDT of hairless mouse skin. PpIX photobleaching curves during a single (A) or light fractionated (B) illumination after topical ALA(■) or BF-200 ALA(□) application.

The surface fluorescence intensity at the start of treatment was not significantly different between the single and light fractionation group treated with ALA or BF-200 ALA or between the ALA and BF-200 ALA groups (t-test, p = 0.73, 0.83 and 0.55 respectively.). The mean light dose delivered in the first light fraction that lead to 48% photobleaching was 2.80±1.12 J/cm^2^ for ALA-PDT and 2.33±0.49 J/cm^2^ for BF-200 ALA-PDT (t-test, p = 0.36). After photobleaching during the first illumination, PpIX fluorescence was accumulated again to the starting value (paired t-test, p = 0.30 and 0.10 for ALA and BF-200 ALA). The photobleaching curves are shown in Fig 6.

#### Changes in vascular area

**Fig 7:**
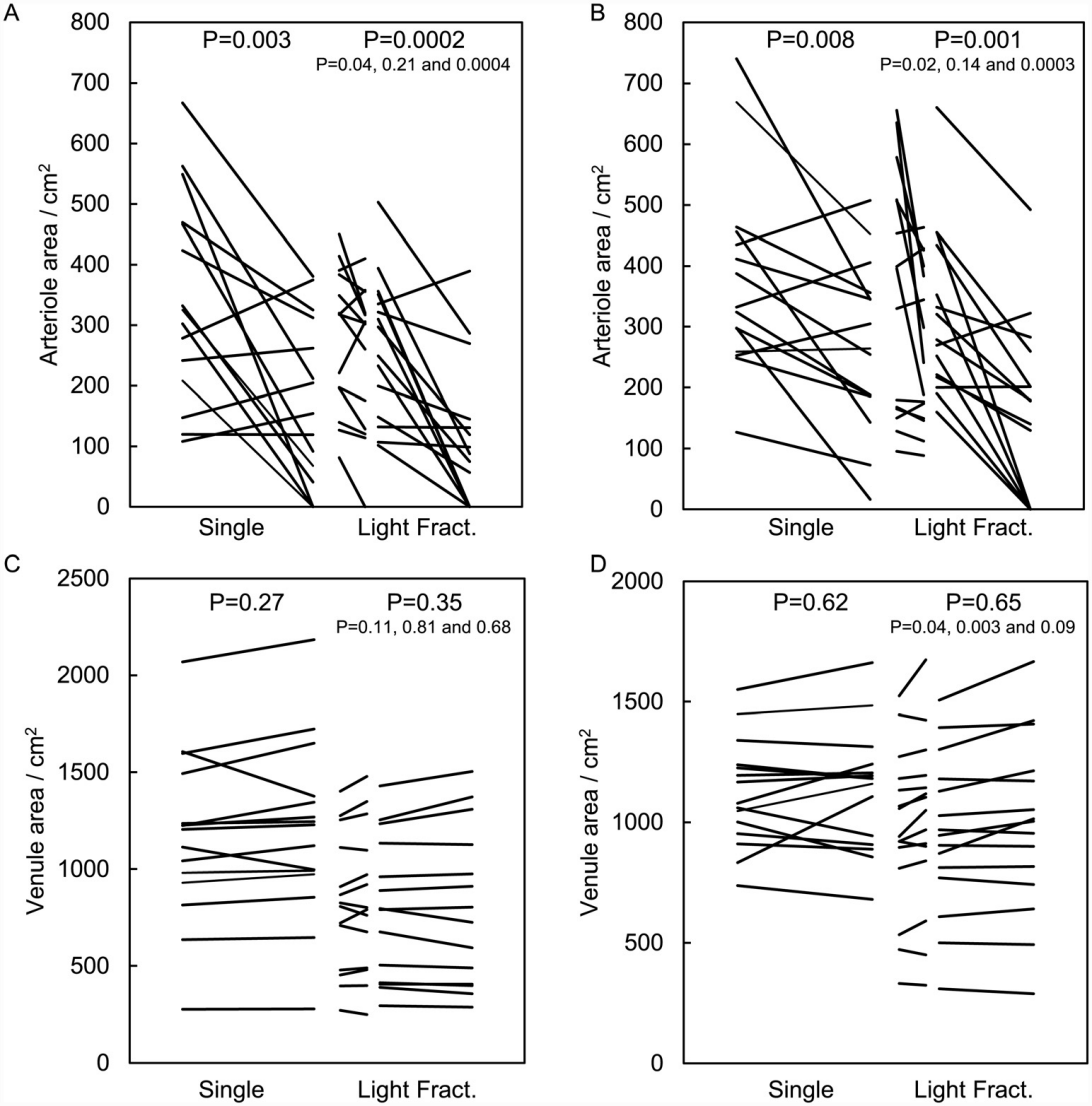
Intra-vital vascular response determined immediately after PDT using the skin-fold chamber model. Change in arteriole (A-B) or venule (C-D) volume during a single or light fractionated illumination after topical ALA (A, C) or BF-200 ALA (B, D) application. Each line represents the result of one location of the maximal three locations in a skin-fold chamber. p values given are paired t tests pre and post illumination.

The change in vascular area during PDT determined in the intra-vital microscopy images collected low in dermis of the skin-fold chamber model is shown in Fig 7A–7D. The arteriole area decreased significantly after PpIX-PDT independent of the illumination scheme used as determined with the paired t-test (Fig 7A and 7B). There was no difference in this response between ALA or BF-200 ALA for a single or a light fractionated illumination (t-test, p = 0.47 and 0.54, respectively). The arteriole constriction seems to be slightly stronger after light fractionated compared to a single illumination after BF-200 ALA PDT than after ALA-PDT although not significant (t-test, p = 0.17 for ALA and 0.06 for BF-200 ALA).

The area of the venules did not change significantly as determined with the paired t-test (Fig 7C and 7D). There was no difference in this response between ALA and BF-200 ALA after a single or a light fractionated illumination (t-test, p = 0.77 and 0.35, respectively). Even when the vascular response to a single or light fractionation scheme is compared for ALA or for BF-200 ALA we found no significant difference (t-test, p = 0.86 and 0.43, respectively). Only with BF-200 ALA light fractionated PDT the area of venules significantly increased during the first fraction, decreased again during the dark period and increased again during the second light fraction. These changes were relatively small and did not result in a significant change of venule area at the end of the treatment (paired t-test, n = 5 animals and 15 locations in total).

#### Vascular integrity

**Fig 8:**
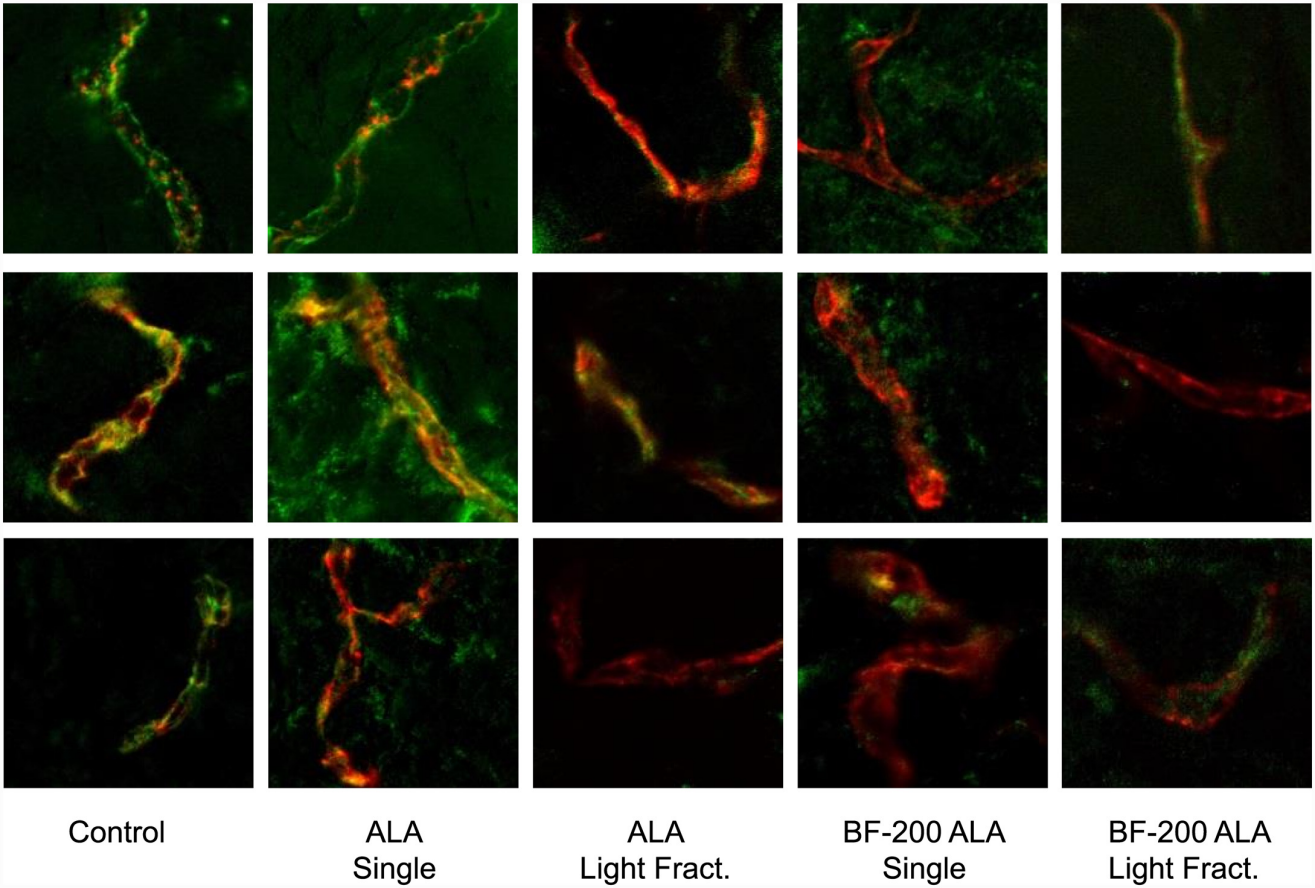
Hairless mouse skin sections collected 1 day post treatment stained for endothelial cells (CD31) and VE-cadherin (CD144). Composite images of anti-CD31 (red) and anti-CD144 (green) fluorescence for control and 4 PDT treated mouse skin samples; ALA-PDT single illumination, ALA-PDT light fractionated illumination, BF-200 ALA-PDT single illumination and BF-200 ALA-PDT light fractionated illumination. For each treatment group three representative unscaled images are shown.

The CD31 fluorescence content in the upper dermis showed no difference between ALA and BF-200 ALA independent of the illumination scheme used. Fig 8 shows a set of three representative composite images of the CD31 and CD144 fluorescence for control and the 4 PDT treated skin samples. The control images show colocalised or closely associated CD31 and CD144 fluorescence. For skin treated with ALA and a single illumination scheme we found less colocalisation and slightly more CD144 fluorescence outside the vasculature. After treatment with ALA and light fractionation we see more loss of CD144 fluorescence and therefore almost no colocalisation. For BF-200 ALA we see the same, i.e. a single illumination results in less colocalisation and more CD144 fluorescence away from the vessels and after light fractionation CD144 seems to be faded, although the intensity of the CD144 overall is lower after BF-200 ALA.

## Discussion

This is the first study to compare ALA and BF-200 ALA. We have previously shown that light fractionation can significantly increase the PDT response in both preclinical and in clinical studies [14–19]. BF-200 ALA, a recently approved nanoemulsion-based gel formulation, is under investigation in several phase III clinical trials and shows slightly lower recurrence rates for AK compared to MAL [28]. In preclinical studies BF-200 ALA was shown to penetrate deeper than MAL in ex-vivo pig skin [30]. The results of the present study show comparable PpIX fluorescence kinetics and deeper PpIX fluorescence at 4 hours in mouse skin for BF-200 ALA and ALA. We also show comparable PDT responses using the visual skin damage score and found a similar increase in effectiveness for the light fractionation scheme for BF-200 ALA and ALA.

The concentrations of ALA used in the present study are different and were chosen to correspond to those used in the clinic; ALA-hydrochloride at 20% and BF-200 ALA contains an ALA concentration comparable to 10% hydrochloride. Several researchers have shown that different percentages of ALA or different volumes of a standard percentage of ALA does not lead to different fluorescence kinetics in normal mouse or human skin [35–36].

All three porphyrin precursors (MAL, ALA and BF-200 ALA) showed PpIX fluorescence extending over the whole epidermis of mouse skin and ex-vivo pig skin after 4 hours of application as we have shown before for MAL and ALA in mouse skin [37]. Our results on ex-vivo pig skin are different from Maisch *et al* despite the use of the same ex-vivo model. They reported PpIX fluorescence only in the upper part of the epidermis after 3 hours and even 8 hours of topical application of MAL and BF-200 ALA, where BF-200 ALA lead to more fluorescence at depth than MAL [28]. This may be explained by the difference in detection methods used. We performed confocal fluorescence microscopy combined with spectral detection and a model based fit of PpIX fluorescence which may be more sensitive than fluorescence microscopic imaging. We detected PpIX fluorescence over the whole epidermal thickness into the dermis. We found that more endothelial cells accumulated more PpIX after BF-200 ALA and ALA compared to MAL. This is in agreement with our previous studies investigating MAL and ALA [27,37].

Using the mouse skin-fold chamber model and intra-vital confocal microscopy we observed differences in the fluorescence intensities at depth in the dermis. We detected significantly more fluorescence after BF-200 ALA application suggesting a deeper penetration compared to ALA. This can be explained by the stabilising effect of the nanoemulsion in BF-200 ALA which is a soybean lecithin-based emulsion gel [38–40].

We note that we were unable to maintain viable mouse skin ex-vivo and that the topical application of ALA, MAL or BF-200 ALA did not lead to PpIX accumulation.

We have demonstrated that light fractionated BF-200 ALA-PDT results in a significant increased efficacy just as we have previously shown for ALA [16]. Specifically, we found no difference between ALA and BF-200 ALA in the response to PDT independent of the illumination scheme. This was true for all of the PDT response parameters we investigated; the visual skin damage score in time post treatment, the rate of photobleaching (that has previously been correlated with ALA-PDT efficacy [41]) and the vascular response as determined by the change in arteriole and venule area, i.e. the vascular volume. We found slight differences in the presence and localisation of VE-cadherin in the upper dermis one day post illumination between ALA and BF-200 ALA. A single illumination results in CD144 fluorescence outside of the vessel after both ALA and BF-200 ALA but the intensity of this fluorescence is lower after BF-200 ALA suggesting there is not only relocation but also loss of VE-cadherin suggesting more vascular endothelial disruption. After light fractionation we see much less CD144 fluorescence compared to a single illumination and we see also less CD144 after BF-200 ALA compared to ALA. In a previous study we only investigated light fractionated ALA-PDT compared to control skin and found a significant loss of CD144 fluorescence [27]. In the present study we have also investigated the distribution and intensity of CD144 fluorescence. The lower intensity of CD144 fluorescence in BF-200 ALA treated skin samples suggest more damage to the vasculature than after ALA.

It is important to consider the wider applicability of the present results obtained in normal mouse skin. While tumours and their vasculature might show different results, the optimization of treatment schemes in this model has previously served as a good predictor of clinical response [18–19]. The results of the present study illustrate the potential of light fractionated BF-200 ALA in clinical translation where ALA and other porphyrin precursors are currently used. We note that the potential benefit of enhanced BF-200 ALA penetration, that could not be investigated in the present study, combined with the benefit of enhanced efficacy using light fractionation could be an important advantage for other skin lesions such as vulval intraepithelial neoplasia and more difficult to treat pre-malignant lesions such as cervical intraepithelial neoplasia or squamous cell carcinoma in the oral cavity.

We have hypothesized that the mechanism behind the increased response to light fractionation is based on a cellular mechanism in addition to the treatment parameters, also the spatial distribution, in particular endothelial accumulation, is important [25]. Cells are sublethally damaged during the first fraction and become more vulnerable to a second light fraction 2 hours later. We have also shown that the dose of the first fraction and the length of the dark interval is important; the dose of the first fraction should not be too high and shortening the dark period results in less effective treatment [16,20–21]. It is important to note that dose in this context refers to the PDT dose, i.e. the dose that leads to singlet oxygen formation, which is influenced by the choice of irradiance, light dose PpIX concentration [25]. While singlet oxygen has a short life time the primary target should be close to the site of accumulation [42]. The current study did not investigate the responses to light fractionated PDT on a subcellular level but there are a two primary targets described in the literature that may be important. The mitochondrial peripheral-type benzodiazepine receptor complex (PBR) is known to be primary target of ALA-PDT and is involved in transport of porphyrins like PpIX across the mitochondrial membrane [43]. A small amount of damage to this receptor may lead to a different location of PpIX accumulation during the dark interval resulting in a different, more sensitive, primary target. Cardiolipin is an unsaturated inner mitochondrial membrane lipid that is considered to be another primary target for ALA-PDT resulting in mitochondria-dependent apoptosis [44]. It is likely that illumination with a first fraction will damage the cardiolipin but not at a level at which the oxidized cardiolipin transfers from the inner to the outer membrane to form the permeable pores that releases cytochrome c. Mitochondria that are damaged trigger the autophagy pathway, and in particular the mitophagy pathway. It would be interesting to investigate why cells are more sensitive to a second illumination 2 hours after a first sub lethal illumination.

Both ALA and BF-200 ALA but not MAL-PDT show significant increased response to light fractionation. The significant lower PpIX accumulation in endothelial cells after MAL application, shown in the present and previous studies, suggests the involvement of the vasculature [27]. In the present study we therefore investigated the vascular responses in various ways after single and light fractionation. Epithelial cells show high fluorescence intensities after topical application but also endothelial cells show PpIX fluorescence to a certain extent. These cells might even be more sensitive to light fractionation due to this lower PpIX concentration. The results of the present study support this hypothesis since there is more arteriole constriction and more loss of VE-cadherin after light fractionation.

As mentioned previously, an optimized response to light fractionated PDT is dependent on the damage induced by the first light fraction, which is influenced by the choice of irradiance, light dose and the PpIX concentration [25]. This means that any combination with other optimization methods for increasing the response to therapy may have a different optimal treatment scheme. For example, the haem cycle can be modulated to result in more PpIX accumulation. Illumination with the same illumination scheme may than result in too much damage in the first light fraction leading to a different subcellular response that stops the cells to become more vulnerable to a second light fraction.

In summary, we compared the temporal and spatial fluorescence distribution and PDT response to ALA-PDT with BF-200 ALA-PDT. Both formulations show similar fluorescence kinetics in normal mouse skin and similar PpIX fluorescence intensities within endothelial cells. Both formulations show significantly increased response to light fractionation compared to a single illumination as shown by the visual skin damage response in mouse skin. There are however small differences. BF-200 ALA showed more PpIX fluorescence at depth in the dermis of living mouse skin compared to ALA suggesting a deeper penetration. Also more loss of VE-cadherin was found one day after BF-200 ALA-PDT compared to ALA-PDT. The current results show the potential of light fractionated BF-200 ALA for clinical translation where ALA is currently used and in more difficult to treat conditions such as vulval or cervical intraepithelial neoplasia or lesions in the oral cavity.

## Supporting Information

S1 Text Porphyrin precursor application. (DOCX)

S2 Text Superficial fluorescence kinetics measurements in mouse skin. (DOCX)

S3 Text PpIX fluorescence distribution and co-localisation with endothelial cells; Fluorescence immunohistochemistry, imaging and analysis. (DOCX)

S4 Text PDT induced vascular response in skin samples 24 hours after PDT; CD31 and CD144 staining, fluorescence imaging and analysis. (DOCX)

## Abbreviations

ALA: 5-aminolevulinic acid
PDT: photodynamic therapy
PpIX: protoporphyrin IX
AK: actinic keratosis
MAL: methyl aminolevulinate
