# Supplementary material for: Light Fractionation Significantly Increases the Efficacy of Photodynamic Therapy Using BF-200 ALA in Normal Mouse Skin
Source: PLoS One. 2016 Feb 12;11(2):e0148850. doi: 10.1371/journal.pone.0148850 (PMC4752243; doi:10.1371/journal.pone.0148850)
Supplement: S1 Text — (DOCX) [file pone.0148850.s001.docx]

**S1 Text. Porphyrin precursor application.** Each porphyrin precursor was applied topically (85 mg/cm^2^) to a 6-7 mm diameter area, covered by a single layer of gauze and occluded using a polythene dressing (Tegaderm, 3M, The Netherlands) to mouse and pig skin for 4 hours unless noted otherwise. In window chambers, precursors were applied to the whole epidermal side of the skin-fold after the removal of the glass spacers. Thereafter, the thin cover slip was replaced to occlude the skin. The vehicle, carboxymethyl cellulose, was applied in control animals. During the application period and between the measurements animals were housed in the dark.
