# Supplementary material for: Light Fractionation Significantly Increases the Efficacy of Photodynamic Therapy Using BF-200 ALA in Normal Mouse Skin
Source: PLoS One. 2016 Feb 12;11(2):e0148850. doi: 10.1371/journal.pone.0148850 (PMC4752243; doi:10.1371/journal.pone.0148850)
Supplement: S2 Text — (DOCX) [file pone.0148850.s002.docx]

**S2 Text. Superficial fluorescence kinetics measurements in mouse skin.** Isoflurane, 2-3 % in oxygen (Abbott, Amstelveen, NL) was used to supplement i.p. anaesthesia. Light from the 532 nm laser was focussed on a 7 mm diameter spot of homogeneous profile on the skin. Excitation light was reflected using a dichroic mirror (535 nm, Omega Optical, Brattleboro, US) and projected onto the skin through a system of lenses. Fluorescence emission from the illuminated area was focussed through the same system of lenses via a dichroic mirror and a 625±20 nm band-pass filter (Melles Griot, Didam, NL) onto a CCD camera (ORCA-ER, Hamamatsu, Japan). A fluorescence reference standard was recorded to correct for the small differences in laser output between experiments. The light dose delivered during each fluorescence measurement was approximately 0.0089 J/cm^2^ at 0.89 mW/cm^2^. Data analysis was performed using ImageJ (v1.47, NIH). The mean fluorescence intensity of pixels within a region of interest was corrected for the dark current, variation in the fluorescence standard and the individual autofluorescence to obtain the PpIX fluorescence intensity from the surface of the skin.
