# Supplementary material for: Light Fractionation Significantly Increases the Efficacy of Photodynamic Therapy Using BF-200 ALA in Normal Mouse Skin
Source: PLoS One. 2016 Feb 12;11(2):e0148850. doi: 10.1371/journal.pone.0148850 (PMC4752243; doi:10.1371/journal.pone.0148850)
Supplement: S3 Text — (DOCX) [file pone.0148850.s003.docx]

**S3 Text. PpIX fluorescence distribution and co-localisation with endothelial cells; Fluorescence immunohistochemistry, imaging and analysis.** Sections were carefully rinsed with PBS and incubated overnight at 4°C with rat anti-mouse CD31-AlexaFluor488 (BioLegend, Uithoorn, NL) diluted 1:200 with 1% BSA/PBS or mouse anti-pig CD31-FITC (AbD Serotec, Uden, NL) diluted 1:100 with 1% BSA/PBS. Sections were re-rinsed carefully with PBS, covered with glycerol (1:3 in PBS) and imaged within 4 hours. All samples were handled in subdued light conditions or darkness where possible to prevent PpIX photobleaching. Fluorescence confocal microscopy was performed using a Zeiss Laser Scanner Microscope 510 (Carl Zeiss B.V., Sliedrecht, The Netherlands) equipped with a 40x Plan-Neofluar objective using a slice thickness of 33 μm. PpIX fluorescence images were acquired first, utilizing 405 nm excitation and spectral detection in lambda mode between 566-727 nm with a 10 nm spectral resolution. Immediately thereafter CD31 fluorescence images were acquired, using FITC or AlexaFluor488, 488 nm excitation and 505-530 nm band-pass detection. Nine sets of images were acquired for each skin sample; 3 sections with 3 images per section widely spaced from each other.

Intra-vital fluorescence images were acquired using a Zeiss Laser Scanner Microscope 510 now equipped with a 10x Plan-Neofluar objective, heated stage and a gas anaesthesia supply unit. Fluorescence images were recorded form three locations within the skin-fold chamber using 514 nm excitation light and spectral detection in lambda mode between 596-681 nm with a 10 nm spectral resolution and an optical slice thickness of 40.2 μm. Corresponding transmission images were recorded for orientation purposes using the same excitation light.

Spectral imaging analysis was performed as described previously using a custom-made script in MATLAB where an autofluorescence basis spectrum was created from spectra collected from control skin and a PpIX basis spectrum was created from spectra collected from highly fluorescent regions [1]. In porcine skin the contribution of pheophorbides to the autofluorescence emission spectrum was carefully taken into account using an additional basis spectrum. The co-localization of PpIX with CD31-positive endothelial cells was investigated in ImageJ where regions of interest were drawn around endothelial cells and the colocalization was calculated using an Intensity Correlation Analysis [2]. Pearson’s correlation coefficient (Pearson’s *r*) was used as a measure of colocalization. The PpIX fluorescence in the lower dermis of the skin-fold chamber was also investigated in ImageJ where regions of interest were drawn around the arteriole wall, venule wall, adipose cells or hair follicles in the corresponding transmission images and superimposed on to the PpIX fluorescence images.

1. Middelburg TA, de Bruijn HS, van der Ploeg-van den Heuvel A, Neumann HA, Robinson DJ. [The effect of light fractionation with a 2-h dark interval on the efficacy of topical hexyl-aminolevulinate photodynamic therapy in normal mouse skin.](http://www.ncbi.nlm.nih.gov/pubmed/24284130) Photodiagnosis Photodyn Ther. 2013;10(4): 703-709.
2. Li Q, Lau A, Morris TJ, Guo L, Fordyce CB, Stanley EF. A syntaxin 1, Galpha(o), and N-type calcium channel complex at a presynaptic nerve terminal: analysis by quantitative immunocolocalization. J Neurosci. 2004;24: 4070-4081.
