# Supplementary material for: Light Fractionation Significantly Increases the Efficacy of Photodynamic Therapy Using BF-200 ALA in Normal Mouse Skin
Source: PLoS One. 2016 Feb 12;11(2):e0148850. doi: 10.1371/journal.pone.0148850 (PMC4752243; doi:10.1371/journal.pone.0148850)
Supplement: S4 Text — (DOCX) [file pone.0148850.s004.docx]

**S4 Text. PDT induced vascular response in skin samples 24 hours after PDT; CD31 and CD144 staining, fluorescence imaging and analysis.** Sections were fixed in acetone for 20 minutes, cellular permeability was increased using 0.5% triton in 1% BSA/PBS for 30 minutes and non-specific staining was reduced using 5% BSA/PBS for 1 hour. Sections were incubated at 4°C overnight with both rat anti-mouse CD31-AlexaFluor647 (Biolegend, Uithoorn, NL) and rat anti-mouse CD144-biotin (eBioscience, Vienna, Austria). After rinsing with PBS the sections were incubated for 30 minutes at room temperature with streptavidin eFluor570 (eBioscience, Vienna, Austria). Sections were rinsed for the last time and covered with glycerol (1:3 in PBS) kept at 4°C to be imaged within 10 days. Fluorescence confocal microscopy was performed using a Zeiss Laser Scanner Microscope 510 (Carl Zeiss B.V., Sliedrecht, The Netherlands) equipped with a 10x and 40x Plan-Neofluar objective.

Per sample we recorded the CD31 fluorescence over the whole length of three sections using the 10x objective, 633 nm excitation and a 650 nm long pass filter. Subsequently, nine sets of images containing a CD31 and a CD144 fluorescence image, were acquired using the 40x objective; 3 sections with 3 images per section widely spaced from each other. CD31 fluorescence images were acquired as with the 10x objective. CD144 fluorescence images were acquired using 514 nm excitation and spectral detection in lambda mode between 540-625 nm. The imaging conditions like gain, excitation power and pixel dwell time were standardized for all samples.

The presence of vessels in the upper dermis is determined in the low magnification images based on the CD31 fluorescence. Image analysis was performed in ImageJ and ROI were drawn around the upper dermis, the threshold was set automatically using IsoData and the CD31 content was determined using Analyze Particles. The colocalisation of CD31 and CD144 was investigated in the high magnification images. Imaging analysis on the CD144 spectral images was performed as described above using basis spectra for autofluorescence and eFluor570. A composite image of CD144 and CD31 fluorescence was created for all sets of images.
